# Involving mosques in health promotion programmes: a qualitative exploration of the MCLASS intervention on smoking in the home

**DOI:** 10.1093/her/cyx051

**Published:** 2017-07-11

**Authors:** R. King, S. Warsi, A. Amos, S. Shah, G. Mir, A. Sheikh, K. Siddiqi

**Affiliations:** 1 Leeds Institute of Health Sciences, University of Leeds, Leeds, UK; 2 Department of Health Sciences, University of York, York, UK; 3 Usher Institute of Population Health Sciences and Informatics, University of Edinburgh, Medical School, Edinburgh, UK

## Abstract

Second-hand smoke (SHS) exposure is high among UK Bangladeshi and Pakistani populations, reflecting higher male smoking prevalence and fewer home smoking restrictions than the general population. The Muslim Communities Learning About Second-hand Smoke (MCLASS) study explored the feasibility and acceptability of implementing SHS education in 14 UK mosques. Religious teachers (RTs) in seven intervention mosques were trained and provided with a culturally appropriate educational package. After the intervention, mosque leaders, RTs and congregants’ experiences and perceptions of the intervention were explored through interviews and focus group discussions. Delivery of the intervention varied across mosques. Facilitators and barriers included: mosque diversity (congregation size, organizational structure, educational activities, women’s role and involvement); degree of trust between researchers and personnel; and views on SHS. Most participants thought mosques’ involvement in SHS health promotion was appropriate, but the perceived importance of SHS differed. We found that a health promotion programme delivered within Islamic religious settings that engages RTs in the process of facilitation, can be acceptable and feasible, but care must be taken to explore the culture and ethos of the institution, including its organizational structure, management committee, RTs and congregation.

## Introduction

Exposure to second-hand smoke (SHS) is an important public health risk accounting for an estimated 600 000 deaths globally each year [1]. In the UK, SHS exposure causes around 12 000 deaths annually, nearly 1 in 10 of all tobacco-related deaths [2, 3]. It is an important cause of ill-health in children who are at risk due to their smaller airways, more rapid breathing and more limited options to remove themselves from SHS exposure than adults [4, 5]. Since the implementation of comprehensive smoke-free legislation in the UK, there have been significant reductions in children’s and adults’ exposure to SHS and consequent health benefits [6–11]. However, SHS exposure still remains highest in disadvantaged groups [12]. These include South Asian-origin communities in whom rates of smoking among men—particularly those of Bangladeshi origin—are higher than those in the general population [13]. For example, a 2008 household survey in a deprived area of the North of England, where nearly half the population was of South Asian origin, found that smoking took place regularly in front of children in 42% of households with at least one smoker [14].

A longitudinal qualitative study evaluating the impact of the smoke-free legislation in England found that this produced positive changes in smoking behaviour including increased quitting attempts and reductions in consumption levels. However, the impact on men of Bangladeshi origin was limited by structural constraints and more pervasive smoking-related cultural values and practices [15]. Reflecting the findings of previous qualitative studies on barriers to smoking cessation among South Asian men [16, 17], the study found that among some Bangladeshi peer groups smoking was a deeply embedded social practice imbued with cultural values relating to hospitality and respect for elders. Although smoke-free legislation had increased awareness about SHS and started to shift social norms around the acceptability of smoking in certain contexts, normal rules governing smoking in the home, even those that were usually smoke-free, continued to be routinely suspended to avoid appearing disrespectful to visiting elders or family members who smoked. The authors argued that there was a need to develop supportive behavioural change interventions in a range of contexts, which would address the specific needs and experiences of ethnic and religious minority communities.

Adopting a settings-based approach to health promotion is recognized as having several strengths including the opportunity to develop interventions, which are tailored to the specific social and cultural context of target populations [18]. Potential settings include religious- and faith-based organizations such as churches, mosques or synagogues, where trusted and respected religious teachers (RTs) and leaders can be involved in the intervention. Moreover, promoting health in minority ethnic groups requires an understanding of both ‘surface’ and ‘deep’ dimensions of cultural sensitivities [19, 20]. The ‘deep’ dimensions, such as religious and sociocultural constructs, help in connecting with the beliefs, values and structures of communities thereby enhancing salience, acceptability and uptake of health interventions [21]. While the potential for involving religious organizations and settings in health promotion has been recognized, most of the evidence on health programmes that take account of a ‘faith dimension’ has come from church-settings in US African American communities [22]. The evidence has been judged to be generally methodologically weak, but indicative of potential benefit. Few studies have explored the feasibility of developing and delivering such programmes in non-church religious settings such as mosques [23].

Almost 92% of people of Bangladeshi- and Pakistani-origin in the UK are Muslims and one-half attend mosques at least once a week. While there is continued debate amongst Muslim religious scholars globally as to whether smoking is *mukrooh* (discouraged) or *haram* (prohibited), many believe that tobacco use conflicts with Islamic teaching, even if not explicitly prohibited. There are several indirect references in the body of Islamic text/literature that are interpreted as a discouragement of its use on the basis of its addictive nature and harm to one’s health [24]. Furthermore, it has been shown that religion is an important influence on the beliefs and attitudes about smoking in Pakistani- and Bangladeshi-origin Muslim communities in the UK [25] suggesting that mosques, given their influential status in UK Muslim communities, could play an important role in helping to address tobacco-related behaviours. However, we found only a few examples in the international literature of RTs in mosques being specifically engaged to deliver broad health promotion interventions, for example on cardiovascular disease prevention [26], or holding one-off health fairs where health professionals provide health education and raise awareness about a range of health issues [27]. We also found some UK health promotion initiatives on smoking cessation with mosques working in partnership with the health services to increase uptake of NHS ‘stop smoking’ services and/or ‘increase quit attempts’ during Ramadan [28]. The study reported here aimed to increase our understanding of the potential role of mosques and RTs in delivering health promotion programmes, in this case a health education intervention aimed at reducing SHS exposure in the home.

### Smoke-free homes (SFHs)

The SFH package was developed in collaboration with Muslim RTs and initially piloted in five mosques in the north of England [29]. It consisted of factsheets detailing key information on smoking, SHS and SFH, guidance and exercises on situating this information within an Islamic context, and a leaflet on smoking and SHS for participants to take away.

A pilot randomized controlled trial called Muslim Communities Learning About Second-hand Smoke (MCLASS) was designed to assess the feasibility of conducting a large definitive trial to evaluate whether the delivery of this educational package by RTs in Islamic settings would reduce non-smokers’ exposure to SHS, as measured by salivary cotinine level [30]. Mosques in the intervention arm were offered the SFH educational package and RTs were trained by a researcher during a site visit on how to use the resource so that they could deliver the package in their setting. Mosques in the control arm did not receive the SFH intervention, but on completion of the trial they were offered the SFH educational package and a detailed guide on training RTs on its use. The detail on findings of the pilot trial in relation to recruitment, effect size, response rates and costs are reported elsewhere [31]. We present the summary of findings of the pilot trial in Box 1.

Box 1: Summary of findings of the MCLASS pilot trial

Recruitment (79%) and retention (100%) rates for clusters were encouraging which reaffirmed our approach of an effective engagement with mosques. Of those eligible, 74% of households participated in the study. The majority of eligible households consented to complete the household surveys; however, the number of those consenting to providing saliva sample was lower. No evidence of a difference between the intervention and control arms in SHS exposure levels and in the secondary outcomes, i.e. proportion of adult smoking, proportion of smokers that reported an intention to quit and proportion of households with smoking restriction was found. However, this evidence is not backed up by formal sample size calculation. The findings of the economic analysis suggest that SFH in these religious settings is a very low cost intervention. Only very modest effectiveness is therefore required to ensure that the intervention is cost-effective.

This article reports the findings of a qualitative study that was embedded within the pilot trial. It focuses on issues relating to the delivery of the SFH package by RTs in mosques and associated religious settings including women’s circles, Qur’an classes and Islamic schools. The research questions were:

1. What are the barriers and facilitators for integrating the SFH educational package into mosque practice?
2. How acceptable is it for RTs to take on a health promotion role?
3. What are the views and experiences of participants regarding the SFH educational package?

## Materials and methods

The experiences and perceptions of those involved in the intervention settings were explored through individual, face-to-face interviews with the mosque chairs (MCs), RTs and recruitment officers (ROs) for the trial, and focus group discussions (FGDs) with women, children and men who regularly attended the mosques and associated religious settings.

The aim was to interview all MCs and the main RT from each of the seven religious institutions included in the intervention arm of the trial. However, only three of the seven MCs were interviewed. Three did not respond to the interview request and one declined. RTs from six of the seven institutions were interviewed, one could not be interviewed without the MC’s consent and he did not respond to requests. All ROs were also interviewed as they had had considerable contact with the religious institutions throughout the intervention period. One FGD was carried out with men who regularly attended one of the mosques; women who attended a women’s circle at a different institution; and children who attended Qur’an classes at a third religious institution (Table I). FGD participants (*n* = 5–10) were recruited through RTs. They were requested to invite participants who reflected diversity in age, socioeconomic background and ethnic affiliation. Participants were given information sheets and provided written consent. Each was given �20 to cover their time and expenses.

**Table I.** Religious institutions and interview and focus group participants

Institution	Description	Interviews conduted	Focus groups
Yellow Mosque	Large mosque in a middle class residential area. Separate building with several rooms and large prayer hall. Another building houses the mosque school, a room for women, a sports hall and other rooms. Congregants mainly South Asian who travel from across the city. Sermons and classes given in Urdu and English	Chair, RT	Women
Brown Mosque	No information	None	
Pink Mosque	Smallest mosque. A small prayer room on a high street. Daily attendees were local Pashtun business men, Friday prayers local male workers. No female provision. One imam from Bangladesh and the other British Asian. Sermons and lessons in Urdu or English	RT	
Blue Mosque	Similar in size and number of attendees as the Yellow mosque. A multi-storey building which includes an after-school school complex. Congregants mainly Arab and North African. The school employs regular teachers and has an accredited curriculum based on age and level of achievement	RT	
Emerald Mosque	A medium sized, two-storeyed building with a main prayer room, located off the high street in a predominantly South Asian neighbourhood. Mosque attendees all male. No separate women’s space. A women’s circle held in a nearby building and girls attend children’s classes in the mosque	Chair/RT^a^	Men
Red School	Provides after-school, summer, half-term, and homework support programmes for school children in a range of areas of Islamic education. Classrooms recently refurbished with AV and computers. Employs qualitified instrutors, follows a set curriculum which is in English	Chair/RT^a^	Children
Purple Mosque	A large mosque located in the centre of the town. Caters mostly to the South Asian community	RT	

a One individual with role of both Chairperson and RT.

Interviews took place in locations selected by interviewees, which were either mosque premises or personal residences, and FGDs took place in the respective institutions**.** Interviews and FGDs were conducted using guides (Table II). The interview guides were structured to allow for open ended responses to questions that had been developed based on the research questions. Interviews and FGDs were conducted in English, Urdu and Punjabi by two experienced qualitative researchers (SW and GM). The interviews with MCs, RTs and ROs and the FGDs with men and children were conducted by a male researcher (SW), whereas the FGDs with the women was facilitated by a female researcher (GM). Both researchers had cultural and linguistic backgrounds that enabled them to communicate and develop rapport with the participants. The interviews and FGDs were recorded, transcribed and translated into English. Transcriptions were anonymized for persons and places to ensure participant confidentiality. Mosques and other religious institutions have been assigned a pseudonym (colours) in the Results section of this article.

**Table II.** Topic guides

Participants	Areas explored
MC Interview	Awareness of project and role in decision to participate Practical delivery of intervention (Did it take place, opinions on project, facilities/difficulties)
RT Interview	Awareness of project and role in decision to participate Practical delivery of intervention (Did it take place, opinions on project, facilities/difficulties) Views on training and support from project Opinions on appropriateness of project (Can people be affected? Were they?)
RO Interview	Experience of recruitment and project implementation (methods used, challenges, and facilities) Perceptions of and relationship with religious institution
FGD	Experience of the intervention Opinions on appropriateness of health promotion in mosques delivered by religious leaders Perceptions on ability to influence others in community Perceptions of impact on behaviour change Smoking practices in home and community

Transcripts of initial interviews were analysed to develop themes for further analytic work [32]. Coding was undertaken manually using an inductive–deductive approach and codes were generated and organized into themes by laying them out and visually arranging them [33]. Emergent codes and themes from further interviews and FGDs were incorporated to robustly describe the experiences and views expressed in the data in response to the key research questions. Fieldnotes from visits to the different institutions were incorporated into the analysis to provide a context for participants’ accounts and consider what role the presence of researchers might play in the nature of responses received. SW translated the interviews and FGDs and developed initial codes and themes. Transcripts, codes and themes were shared with AA and RK for the initial three interviews. While there was no disagreement among the researchers on the initial codes and themes used for analysis, minor modifications and additions were made to broaden the themes.

Ethical approval has been obtained from the local NRES Committee and the University of York Health Sciences Research Governance Committee.

## Results

### Integrating the SFH educational package: barriers and facilitators

The interviews revealed variations in the delivery of the intervention (Table III). Of the six institutions for which we could gather information, five delivered the SFH package via sermons and/or school assemblies (there was no information on the Brown mosque, and the Blue mosque did not seem to deliver the intervention), but some also delivered it through smaller classes or circles, and the Red School incorporated the messages and activities into its curriculum. It was difficult to determine how many times the package was used. The RTs at Pink and Emerald Mosques were able to give ranges of times, whereas the RT at the Yellow Mosque was unable to provide a concrete response, stating:

*“You see, I have so many topics of my own to convey *[*for the sermons*]*, so naturally I can never address all of them. So maybe once or twice a month I’ve told people”* (RT Yellow Mosque)

**Table III.** RTs’ reported implementation of the SFH intervention

Religious institution	Intervention settings			
	Sermon/assembly	Lecture/class	Other	Frequency of messaging
Yellow Mosque	✓	✓	�	Numerous
Brown Mosque	?	?	?	?
Pink Mosque	✓	�	�	2–3 times
Blue Mosque	�	�	�	0 times
Emerald Mosque	✓	�	�	15–20 times
Red School^a^	✓	✓	�	3 times
Purple Mosque^a^	✓	✓	✓	Numerous

a Both institutions were implementing other projects dealing with the issue of active and/or passive smoking.

The RT’s vagueness was likely related more to the system of instruction at the mosque than to not having used the package. This contrasts with Purple Mosque where the RT used the package in sermons and children’s classes:

*“Now this is your project, but we experimented with it! … Three times I spoke in the sermons. In the classes, I talked about it incrementally, about two days a week, [and] we did one or two activities and we had three assemblies for sure. And aside from that we kept on doing things like the posters on the harms [of smoking].”* (RT Purple Mosque)

Several barriers and facilitors to the delivery of SFH were identified. These related to the diversity of religious institutions in terms of the relative sizes of congregations and mosque staff, location and infrastucture, the demographics and ethnic origin of congregrants, the background of imams and other RTs, languages used and the range and scale of activities held in the mosque (Table I). Some mosques were community hubs and included a range of facilities such as schools, social centres and leisure facilities, and had large numbers of attendees. Others were more local, neighbourhood-centred institutions, which also provided limited religious classes for children. Finally, there were small *musallahs* (prayer rooms) catering for people working in the area, which did not support a local community.

This was paralleled by the diversity of RTs and congregants. While the mosques included in this research catered primarily for specific ethnic groups from South Asia or the Middle East, research participants said they were attended by ethnically and linguistically diverse groups of people. There was a further diversity in religious approach among the mosques based on the dominant religious sect within the congregation, which could affect the kinds of educational activities provided through the mosque and attitudes toward gender segregation. The mosques were also linguistically diverse, making English one of the primary languages of communication among congregants, particularly between attendees of different generations. Communities also differed in this respect: while older attendees in some mosques might have had very limited fluency in English, elsewhere older participants tended to be multi-lingual and able to switch between English and other languages such as Urdu, Punjabi, Pashto and Dari. Younger generations tended to either be monolingual in English or also have comprehension of one other language spoken in the community.

The type of mosque and its role in the community impacted on both the mosque staff’s capacity to deliver the SFH educational package and the breadth of potential participants. The dominant school of thought within the congregation also could affect the kinds of educational activities provided and attitudes towards women’s participation.

A related issue which proved challenging was the diversity in mosques’ administrative and power structures. Some mosques had active and engaged committees, where the RT acted on the instruction of the committee members. In other mosques, the RT was more independent and had more authority in determining lessons and educational activities. For example, the Purple mosque RT operated independently of the mosque committee. When asked if the committee had decided on whether to take part in the project he stated:

*“I took the decision myself. And as you know, I’ll repeat, I’m in charge of the educational arm [of the local branch of a national religious group] and nobody else can interfere in these matters. Whatever there is to be done on Fridays I do it myself.”* (RT Purple Mosque)

In other cases the RT operated only with the permission of the committee. Emerald Mosque RT explained how sometimes even if an RT was capable, nothing could be done without the consent of the mosque committee:

*“… the imam has the most influence on the public, but sometimes the imam cannot do this kind of work against the wishes of the mosque committee. If the committee members help, then the imam is everything. The imam can do a lot… For this reason, please focus on the committee first, then on the imam.”* (RT Emerald Mosque)

A similar view was echoed by an RO who highlighted the importance of building trusting relationships with relevant mosque personnel:

*“You need to develop some kind of rapport or kind of relationship with – trusting relations – with imams [so they know] that you’re embarking on this work way before you start the project, so that the people should know you, that who you are, and what you’ll be doing, [and] how you will do it. This thing is very important.”* (RO1)

Mosques differed in terms of the level of organization of their activities. A lack of organization within the mosque created problems at many sites. In contrast, Blue Mosque’s very well-organized educational curriculum, which was planned a year in advance, made it difficult to incorporate the intervention in a short time frame:

*“Because, it was … last month I met [the Research Officer], I think it was last month [October]. And … [RO] wants [the intervention delivered] for the end of December and I would have wanted an even further deadline as we’re starting the scouts and I’m a leader in the scouts and … more.”* (RT Blue Mosque)

In contrast, Yellow Mosque, which delivered SFH to children, had a more *ad hoc* model where the RT determined the content of lectures and classes. Red School and Purple Mosque RTs did have a pre-planned curriculum, but were able to deliver the intervention, suggesting it was easier in a school setting to deliver the intervention through pre-planned assemblies. Purple Mosque RT was also head of a separate educational institution and therefore possibly had more skills to modify and incorporate new material into the mosque curriculum.

This links with the last barrier/facilitator, which emerged, the difference in RT skills and capacities. The RTs had varying levels of fluency in English, educational backgrounds and skill sets with regard to management and use of educational technology. For example, Red School and Purple Mosque RTs suggested that the SFH materials could be developed to include additional information and activities to engage pupils of different ages. In contrast, Emerald Mosque RT acknowledged that he needed additional training to deliver the educational methods used in SFH.

*“… they could have given us a training of all the possible activities we could have done under this project, like “you can do such and such activities.” Now, they gave us a piece of paper with the activities, but until it is not shown practically how to do it … This training was not given.”* (RT Emerald Mosque)

The Purple Mosque RT presented the issue of RTs’ varying capacities and interest in terms of age and immigrant generation.

*“… if you say to Urdu- or Punjabi-speaking ulema* [scholars] *“Go do this and that,” they will not listen to you. You need active people, youngsters! You should go target them in the mosques. I’m young myself, and active and I can do these things, [like] operating a computer, making posters, courses, etc. They can’t do these things; they’re behind in these areas.”* (RT Purple Mosque)

However, in contrast to Purple Mosque RT’s view, one RO reported that the imam at Yellow Mosque, a Punjabi and Urdu speaking first-generation immigrant, though not initially interested in the project or cooperative, became more interested after the training when he understood more about SHS. Emerald Mosque RT also indicated that another reason RTs might not be as involved was because they would have to dedicate “*special time*” to the project without any incentive.

### The acceptability of RTs taking on a health promotion role

Participants in both the interviews and FGDs were unanimous in their view that RTs were acceptable conveyors of health information. As was expressed in the men’s FGD:

*“It’s an imam’s job anyway, Islamically, to tell people about smoking or any evil in the society”* (FGD-Men)

Most participants also agreed that RTs were regarded as authoritative and respected figures whose opinions were influential. However, several RTs indicated that their authority on science and health related topics could benefit from being supported by health professionals speaking to mosque attendees alongside them.

Responses to questions on the appropriateness of the mosque as a space for receiving health-related information revealed a more complex picture. The men’s FGD participants said that, while leaving leaflets at the mosque would be acceptable, using the mosque as a place to give health information via a project like SFH was not as well accepted:

*“ … when you come to the mosque, you want to pray, you know? And [its’] a place of worship really. And you don’t want to come here and do other things you know? You want to escape from these things you see.”* (FGD-Men)

These participants, from Emerald Mosque, regarded the mosque as purely a place where people came to pray rather than a community centre. This view was also shared by Pink Mosque RT. Others participants including Purple Mosque RT, Emerald Mosque RT, and Yellow Mosque chairperson all expressed the view that while there were individuals who saw the mosque as a place only to pray or to conduct sectarian politics, the mosque should be cultivated to serve as a community centre, not least because of the community investment in the mosque.

*“Millions of pounds are spent on building mosques, so the Muslim community should take as much benefit from them as they can. What are these millions of pounds spent just to perform ablutions and pray? That’s a big question for Muslims. Use them for your education; there should be social education. The Muslim community is most in need for education on health. The Muslim community is known as a sick community in this country as they use unhealthy food, there’s smoking, and other issues. All of these things must be addressed and the mosque should take the lead.”* (RT Emerald Mosque)

The view expressed by most of the ROs was that while the mosque could be a place where the community gathered, it was not always the case and that more people could be reached, and engaged more effectively, through schools and other community centres.

*“Maybe just not using the masjids (mosques) … and doing it in community centres instead … Where there’s a bit more rapport … with people and they understand why we’re doing it [the project] … [Community centres] understand community, but also they understand the social aims of what we’re doing. Whereas the masjids don’t always.”* (RO1)

The RO then qualified this statement, saying that mosques could be more amenable to working on health issues if the committee members were younger. This comment echoed Purple Mosque RT’s opinion that younger mosque personnel are more engaged and open to doing such projects, though some of the mosque personnel who were actively involved in SFH were not the younger members of the mosque administration. The children’s FGD participants indicated that while mosque and religious institutes like Red School were good channels for disseminating health information, other avenues such as after-school clubs and community centres would also be appropriate and could have the added value of getting parents involved.

### The acceptability of education on SHS and the SFH project

There was a striking contrast in the opinions expressed by the children’s and women’s FGD participants and those of the men’s FGD participants about both the SFH intervention and the importance of smoking and SHS more generally. Participants in the children and women’s FGDs were very supportive of the project and indicated not only that they thought it was an important issue, but that several of them had tried to change their family members’ smoking habits. They demonstrated an awareness of the difference between SHS and active smoking, how non-smokers were affected by SHS exposure, and ways to address this in the home. Participants specifically mentioned not smoking in front of children, isolating smoking from the house, and smoking only outside the house.

*“Secondhand smoking is also a bad thing because when the person stops smoking and leaves, … the bad things which comes out of the cigarette will be still in the air and it could be in the air for more than four or five hours.”* (FGD-Children)

Among the women participants, there was a sense that while one might not be able to change a smoker’s habits, people had the responsibility to try. It was generally agreed that smokers should be engaged with in a “good way” so as not to alienate them. Two women reported having persuaded male family members to either quit smoking or start smoking outside the home. Another woman related how when she heard about SHS in the women’s circle she raised the issue with her son-in-law, who responded by smoking secretly in the bathroom and then spraying air freshener to cover the smell. She described how once, when she went to his house, she tried indirectly to embarrass him:

*When I went [to the bathroom], I said: “Who was smoking here?” My daughter said: “you know who smokes right, so why are you making a fuss?” I told her that my reason for making a fuss is so that he should be embarrassed … [I] said “I don’t want to come here, even if he is smoking in the bathroom or wherever, there’s the smell right?”* (FGD-Women)

In contrast, participants in the men’s FGD were not interested in discussing SHS. These participants attended Emerald Mosque where the intervention had been delivered only through the Friday sermons. Throughout the discussion, the participants were keen to emphasize how unhappy they were that the project had been carried out in a mosque. They showed a general awareness of SHS, but when pressed to explain what messages had been delivered in sermons one participant responded curtly:

P1: *“We just heard the same news, you know, don’t smoke, smoking [is] bad for you.”*

P2: *“Yeah, and can [affect] the others in your house”*

P1: *“But what we were thinking, “Why,” it’s already been told really, the information*

It’s been recycled and recycled and recycled.”

There was a perception that education of this type would be ineffective either because mosque attendees didn’t smoke or, if they did, were too set in their ways to change:

*“The people here are old, they already done everything that they need to do. But you need to get to the new generation, the younger generation in school, in nursery … Due to all the information, government information, most of the youth don’t smoke, and if there’re people who do smoke, they do in a hidden way.”* (FGD-Men)

The comment that if people smoked they would try to hide it, suggested that smoking in these communities might be seen as a social taboo. Indeed, they were insistent that neither they nor anyone in their family smoked. A younger man, who became irate at the discussion, exclaimed:

*“ … no-one smokes in our family and even if they did, they won’t dare smoke in front of us, because we’ll beat the crap out of them”* (FGD-Men)

In addition, they stated that did not feel that smoking was an important issue in their community when compared to substance abuse problems.

“*Yeah, this is an area that, when they smoke, they don’t smoke cigarettes. They smoke cocaine, heroin, yeah? So that’s the – smoking is the least of the problem*” (FGD-Men)

There was also a perception that those who smoked did so because of stress from family, societal or governmental structures and distrust. One man described at length how his daughter’s abusive relationship and subsequent loss of her children to the father via social services led to her starting to smoke and how there were other women in the same situation.

## Discussion

This study is one of the first to explore the potential of mosques and associated fora such as women’s circles, Qur’an classes and Islamic schools in the UK as settings for health promotion programmes. The study identified a number of key facilitators to programme delivery including: varied and sufficiently flexible sessions into which the intervention activities could be incorporated; systems for involving women and children in mosque activity; trust in those promoting the intervention; the independence of RTs to make a decision about delivery or otherwise, support of the MC, and RTs with appropriate skills and motivation to engage with the intervention. It was clear, however, that it was difficult to deliver the intervention in a standardized way across diverse settings and that the details of context mattered a great deal in terms of facilitators and barriers.

The findings of this study suggest that, whilst mosques can be acceptable settings for delivering health promotion programmes, this view was not unanimous. FGD participants agreed that RTs are authoritative and respected figures, although some suggested that collaboration with public health professionals could further legitimize the health promotion intervention. Furthermore, women and children responded favourably to the SFH educational package and some reported trying to implement smoking restrictions in their homes, but male FGD participants were critical of the intervention. Diversity in attitudes towards acceptability of the mosque as a setting for SFH delivery amongst male respondents in the study suggests a need for further research. Within this, it could be useful to explore whether demographic characteristics or smoking status of respondents influences attitudes.

A strength of this study was the involvement of both male and female researchers who had cultural and linguistic backgrounds that enabled them to develop communication and rapport with the study participants. However, the study was limited insofar as it explored the views of a small number of RTs and congregants from Muslim communities in three cities in the UK. Moreover, not all mosques and intended participants agreed to participate in this qualitative enquiry and it would have been preferable to access more participant views in order to maximize the variability of the sample. Similarly, due to resource constraints, only three FGDs were conducted and there may have been different responses had it been possible to conduct FGDs with congregants from each participating institution. Despite these limitations, the study has yielded useful information on engaging mosques and RTs in health promotion programmes. It also suggests gender specific attitudes towards the incorporation of SHS education into mosque settings, but care should be taken with the interpretation of this finding, due to the limited sample size.

Recent studies have explored mechanisms for adapting health promotion interventions for minority ethnic communities. A systematic review of interventions designed to reduce coronary heart disease amongst minority ethnic communities in the US and the UK identified five principles to consider when developing a successful behavioural intervention for minority ethnic communities [34]:

1. Use community resources to publicize the intervention and increase accessibility;
2. Identify and address barriers to access and participation;
3. Develop communication strategies which are sensitive to language use and information requirements;
4. Work with cultural or religious values that either promote or hinder behavioural change;
5. Accommodate varying degrees of cultural identification.

When the intervention is assessed against these principles, it is clear that adherence is strong. For example, mosques, women’s circles and Qur’an classes are community resources and accessibility to the intervention was increased, in part by addressing barriers particularly around gender-appropriate interactions. Appropriate communication strategies were utilized through the production of materials and the delivery of training in Urdu and Bengali. A critical component of the intervention design explicitly aimed to accommodate varying degrees of identification with relevant cultural and religious values by encouraging RTs, through training, to situate public health messages within religious discourse and teaching appropriate to their particular setting and congregation. Despite this incorporation of the key principles during development of the intervention, there was a lack of consensus amongst participants around the acceptability of the approach during its implementation, reflecting existing evidence on implementing culturally adapted interventions in this population [35]. This suggests the need for further research to unpack the most effective ways to adapt education on SHS for diverse groups within Muslim populations, as well as further research to understand if and how to best engage with mosques in health promotion campaigns. The need for further evidence echoes the findings of a recent evidence synthesis, which found that there is a lack of evidence on how best to adapt smoking cessation programmes for ethnic minority groups [23].

In conclusion, this study showed that there are challenges in setting up and implementing health promotion programmes in mosques, and a better knowledge of these challenges may help to understand how best to engage mosques in such programmes. These relate, in particular, to the need to undertake rapid, but in-depth contextual analysis of each setting prior to implementation. As discussed there is diversity in the size, location, and administrative and instructional infrastructure of mosques in the UK. This was paralleled by the diverse backgrounds RTs and congregants and this diversity is relevant to implementation of the SFH. Our experience emphasizes the need for building a trusting relationship with mosques by exploring their infrastructure and activities, internal dynamics, administrative structure, and key committee members and personnel in order to tailor the engagement and specifics of the intervention package from the outset.

When developing health promotion programmes, there are always tensions between the requirement to deliver at scale and the need to tailor programmes in order to increase access. Whilst we support the five principles identified by Netto *et al*. [34, see also 20] when developing interventions for minority ethnic communities, we also stress that minority ethnic communities cannot be viewed as homogenous and that further exploration may be required to understand the most effective delivery approach that takes into account diversity within minority ethnic and faith groups.

Finally, this study speaks directly to concerns about addressing health inequalities amongst minority ethnic groups [36, 37]. It shows that a health promotion programme delivered within Islamic religious settings and which engages RTs in the process of facilitation, can be acceptable and feasible, but care must be taken to explore the particular dynamics of the religious institution, its MC, RTs and congregation.
